# Differences in Tau Seeding in Newborn and Adult Wild-Type Mice

**DOI:** 10.3390/ijms23094789

**Published:** 2022-04-26

**Authors:** Isidro Ferrer, Pol Andrés-Benito, Paula Garcia-Esparcia, Irene López-Gonzalez, Diego Valiente, Mónica Jordán-Pirla, Margarita Carmona, Julia Sala-Jarque, Vanessa Gil, José Antonio del Rio

**Affiliations:** 1 Department of Pathology and Experimental Therapeutics, University of Barcelona, Feixa Llarga sn, 08907 Hospitalet de Llobregat, Spain; pol.andres.benito@gmail.com (P.A.-B.); p.garcies@gmail.com (P.G.-E.); lopez.gonzalez.irene@gmail.com (I.L.-G.); diego.valiente.cerro@outlook.com (D.V.); mjordanpirla@gmail.com (M.J.-P.); mcarmona@idibell.cat (M.C.); 2 Bellvitge Biomedical Research Centre—IDIBELL, Feixa Llarga sn, 08907 Hospitalet de Llobregat, Spain; 3 Network Centre of Biomedical Research of Neurodegenerative Diseases—CIBERNED, Institute of Health Carlos III, Feixa Llarga sn, 08907 Hospitalet de Llobregat, Spain; 4 Molecular and Cellular Neurobiotechnology, Institute of Bioengineering of Catalonia, Barcelona Institute for Science and Technology, Parc Científic de Barcelona, Baldiri Reixac sn, 08020 Barcelona, Spain; jsala@ibecbarcelona.eu (J.S.-J.); vgil@ibecbarcelona.eu (V.G.); jadelrio@ibecbarcelona.eu (J.A.d.R.); 5 Department of Cell Biology, Physiology and Immunology, Faculty of Biology, University of Barcelona, Baldiri Reixac sn, 08020 Barcelona, Spain; 6 Centro de Investigación Biomédica en Red Sobre Enfermedades Neurodegenerativas (CIBERNED), 28031 Madrid, Spain

**Keywords:** tau seeding and spreading, newborn, Alzheimer’s disease, thalamus

## Abstract

Alzheimer’s disease (AD) and other tauopathies are common neurodegenerative diseases in older adults; in contrast, abnormal tau deposition in neurons and glial cells occurs only exceptionally in children. Sarkosyl-insoluble fractions from sporadic AD (sAD) containing paired helical filaments (PHFs) were inoculated unilaterally into the thalamus in newborn and three-month-old wild-type C57BL/6 mice, which were killed at different intervals from 24 h to six months after inoculation. Tau-positive cells were scanty and practically disappeared at three months in mice inoculated at the age of a newborn. In contrast, large numbers of tau-positive cells, including neurons and oligodendrocytes, were found in the thalamus of mice inoculated at three months and killed at the ages of six months and nine months. Mice inoculated at the age of newborn and re-inoculated at the age of three months showed similar numbers and distribution of positive cells in the thalamus at six months and nine months. This study shows that (a) differences in tau seeding between newborn and young adults may be related to the ratios between 3Rtau and 4Rtau, and the shift to 4Rtau predominance in adults, together with the immaturity of connections in newborn mice, and (b) intracerebral inoculation of sAD PHFs in newborn mice does not protect from tau seeding following intracerebral inoculation of sAD PHFs in young/adult mice.

## 1. Introduction

Alzheimer’s disease (AD) and tauopathies are neurodegenerative diseases with abnormal accumulation of hyper-phosphorylated tau in neurons and glial cells [1,2]. All of them are disorders characterised by selective cellular vulnerability and stereotyped patterns of disease progression of variable complexity, as exemplified in AD [3,4,5,6,7,8,9,10,11,12,13,14,15,16,17,18]. Tau pathology is an initiating factor in sAD [19]. The formation of abnormal protein aggregates in particular cell types, and the progression of the disease, are active and very complex processes involving several steps [20,21,22,23,24,25,26]. In addition to specific neuronal, glial, and regional vulnerability, tau transmission from one neuron to another may occur trans-synaptically [21,24,25,27,28,29,30,31]. Free release of tau to the extracellular space and vesicle-associated tau exocytosis are other mechanisms of tau secretion [21,24,32,33,34,35,36]. Tau can also be transmitted through tunnelling nanotubes which are actin-based nanotubular channels that connect one cell to another [37]. This latter mechanism may apply particularly to glial cells. 

A curious situation is the extremely rare occurrence of tau pathology in children, despite the active tau phosphorylation during brain development [38,39]. The reason for the underlying resistance to tauopathy in children is not known. Moreover, the initiation of tau pathology in transgenic mice bearing tau mutations, including P301S transgenic mice which express the T34 isoform of microtubule-associated protein tau with one N-terminal insert and four microtubule-binding repeats encoding the human P301S mutation, all driven by *prnp* promoter, always occurs in young/adult animals, never in newborns [40]. 

Tauopathy can also be experimentally induced following intracerebral inoculation of tau species in mice under appropriate conditions. Thus, intracerebral inoculation of pre-formed synthetic tau fibrils in transgenic mice expressing human mutant tau induces tau pathology in connected brain regions [41,42,43,44]. Seeding and spreading of abnormal tau also arises following inoculation of brain homogenates from P301S transgenic mice, sporadic AD (sAD), and other tauopathies into the brain of transgenic mice overexpressing human 4Rtau or human mutated tau [29,45,46,47,48]. Tau seeding and propagation may also occur in WT mice following intracerebral inoculation of sarkosyl-insoluble fractions obtained from sAD and various tauopathies [26,49,50,51,52,53,54,55]. Therefore, intracerebral inoculation of tau at different developmental stages would afford us the opportunity to learn about age-dependent vulnerability to developing tau pathology.

The present study was geared to learn about the (i) differences in the vulnerability of tau seeding between newborn and young/adult mice aged 3 months following thalamic inoculation of sarkosyl-insoluble fractions of sAD, and (ii) possible protective effects of brain inoculation of sarkosyl-insoluble fractions of sAD homogenates at the age of newborn in mice re-inoculated with the same fractions at the age of three months. To these ends, and to avoid differences linked to particularities of different inoculums, all the animals were inoculated with the same sAD brain homogenate and processed in the same way.

## 2. Results

### 2.1. Tau during Normal Brain Development (Group 1)

#### 2.1.1. Tau Species as Revealed by Western Blotting 

**Figure 1:**
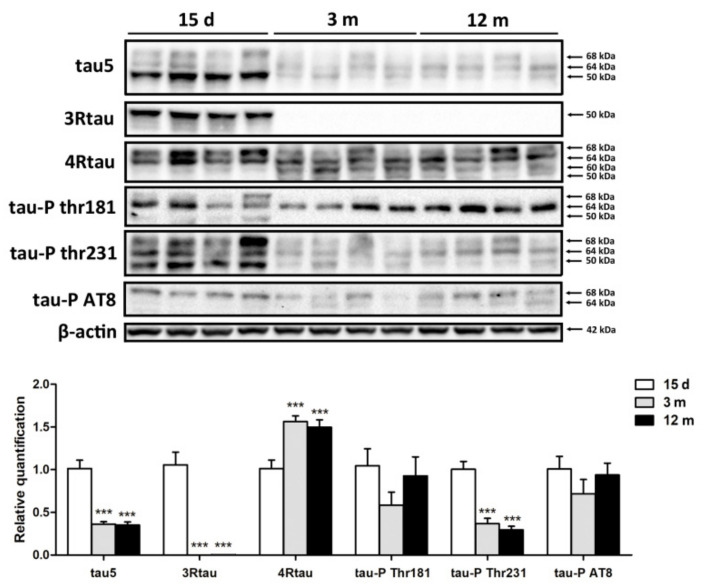
Tau expression in the developing and young/adult brain at the time-points of 15 days, 3 months, and 12 months (4 animals per group) as shown in Western blots of total brain homogenates. Total tau levels are higher at 15 days than at 3 months and 12 months. 3Rtau levels are high at the age of 15 days but decrease markedly at 3 months and 12 months. 4Rtau is expressed in mice aged 15 days, 3 months, and 12 months, but the band pattern differs in mice aged 15 days when compared with young/adult mice. Tau phosphorylation at Thr231 is more marked in mice aged 15 days than in mice aged 3 months and 12 months, but the levels of tau phosphorylation at Thr181 and Ser202/Thr305 (antibody AT8) are similar in the three groups. β-actin was used as a marker of protein loading. Unpaired *t*-test compared 3 months and 12 months with 15 days: *** statistically significant *p* < 0.001.

Western blots of total brain homogenates showed high levels of a wide band of tau-5 with a molecular weight of from 50 kDa to 68 kDa in mice aged 15 days. The expression levels of tau-5 were reduced in mice aged 3 months and 12 months. A similar pattern was observed in Western blots incubated with anti-3Rtau antibodies. High levels of 3Rtau of about 50 kDa were identified in mice aged 15 days. 3Rtau levels decreased in mice aged 3 months and again in mice aged 12 months. The expression of 4Rtau differed from the others. Two bands of 68 kDa and 64 kDa occurred in mice aged 15 days, but three main bands of 68 kDa, 64 kDa, and 60 kD, together with a weaker band of about 50 kDa, were visible at the age of 3 months and 12 months. Tau phosphorylation also differed with age. Tau in mice aged 15 days was mainly phosphorylated at Thr231, to decrease in mice aged 3 months and 12 months. However, the levels of phospho-tau at Thr181 and Ser202/Thr305 (antibody AT8) were similar in the three age groups (Figure 1). Quantification of Western blots is shown in the diagram in Figure 1. The levels of Tau 5, 3Rtau, and phospho-tau Thr231 were significantly higher in mice aged 15 days when compared with mice aged 3 months and 12 months, *p* < 0.001. In contrast, the levels of 4Rtau were significantly higher in mice aged 3 months and 12 months when compared with 15-day-old mice, *p* < 0.001. 

#### 2.1.2. Tau Immunohistochemistry 

Tau immunohistochemistry reproduced the pattern observed in Western blots. Antibodies against Tau5, 3Rtau, and 4Rtau showed a diffuse pattern in the neuropil, which was more marked in mice aged 15 days when compared with mice aged 3 months and 12 months. The antibody anti-tau Thr231 decorated the cytoplasm and dendrites of neurons in young adults, whereas the antibody AT8 stained the nuclei at every age. Deposits of tau were not found in the cytoplasm of neurons or glial cells at any age.

**Figure 2:**
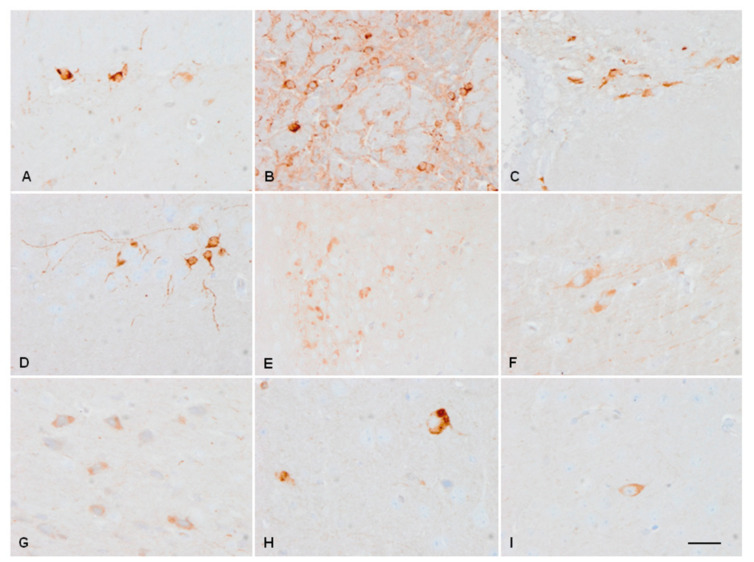
3Rtau-immunoreactive cells persist in the adult murine brain in the internal polymorphic layer of the dentate gyrus (**A**), olfactory bulb (**B**), and a periventricular layer of the lateral ventricle (**C**). Scattered positive neurons are also found in the entorhinal cortex (**D**), periventricular hypothalamus (**E**), nuclei of the basal forebrain (**F**), and thalamus (**G**); very rarely, 3Rtau-immunoreactive neurons are encountered in the amygdala (**H**) and cerebral cortex (**I**). Paraffin sections, lightly counterstained with hematoxylin, bar = 35 µm.

Although 3Rtau decreased and 4Rtau was predominant in the young/adult murine brain, selected neuronal subpopulations expressed 3Rtau in the young/adult brain. 3Rtau-immunoreactive neurons were localised in the inner layer of the granule cells in the dentate gyrus, olfactory bulb, and periventricular layer of the lateral ventricles. In addition, a few 3Rtau-immunoreactive neurons were found in the entorhinal cortex, periventricular hypothalamic nuclei, basal forebrain, and thalamus; very rarely, 3Rtau-immunoreactive neurons were also encountered in the amygdala and cerebral cortex (Figure 2).

### 2.2. Human Sample: Characterization of Sarkosyl-Insoluble Fraction

**Figure 3:**
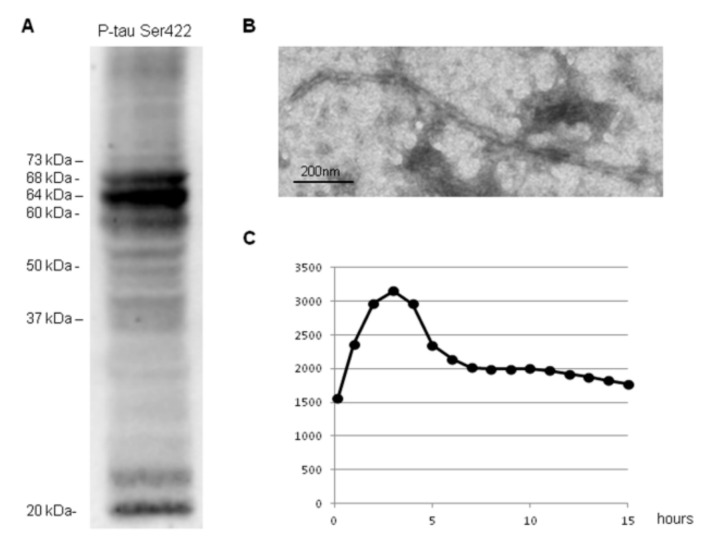
Characteristics of sarkosyl-insoluble fraction from sAD. Western blotting processed with anti-phospho-tau Ser422 (P-tau Ser422) reveals three bands of 68 kDa, 64 kDa, and 60 kDa, a weak upper band of 73 kDa, and oligomeric smears, in addition to several bands of about 50 kDa, between 30 kDa and 40 kDa, and lower bands of truncated tau at the C-terminal, one of them of about 20 kDa (**A**). Transmission electron microscopy of fibres in AD sarkosyl-insoluble fraction showing typical paired helical filaments, bar = 50 nm (**B**). Thioflavin T incubation of sarkosyl-insoluble fractions shows increased fluorescence with time, indicating amyloid fibril formation (**C**).

The characteristics of the patient with sAD are detailed in the section Material and methods. Western blotting of the sarkosyl-insoluble fraction processed with anti-phospho-tau Ser422 antibody revealed three bands of 68 kDa, 64 kDa, and 60 kDa, together with a low upper band of 73 kDa, several bands of about 50 kDa, several bands between 30 kDa and 40 kDa, and two lower bands of truncated tau at the C-terminal, one of which was of about 20 kDa, as well as a smear of higher molecular weight represented tau oligomers (Figure 3A). TEM of the same sarkosyl-insoluble fraction revealed the presence of typical paired helical filaments (Figure 3B). Incubation of sarkosyl-insoluble fraction with thioflavin showed increased fluorescence peaking at three hours and a decrease thereafter (Figure 3C), indicating the presence of amyloid fibrils. 

Western blotting of sarkosyl-soluble fractions stained with anti-3Rtau and anti-4Rtau antibodies showed bands at the expected molecular weights of the six main tau isoforms present in the brain (between 68 kDa and 60 kDa); a lower band of about 37 kDa was also identified with anti-3Rtau antibodies. No bands were stained with anti-phospho-tau Ser422 antibodies. TEM of sarkosyl-soluble fractions revealed no fibrillar structures (data not shown).

### 2.3. Inoculation of Sarkosyl-Insoluble and Soluble Fractions from sAD into the Thalamus of Newborn WT Mice (Group 2)

**Figure 4:**
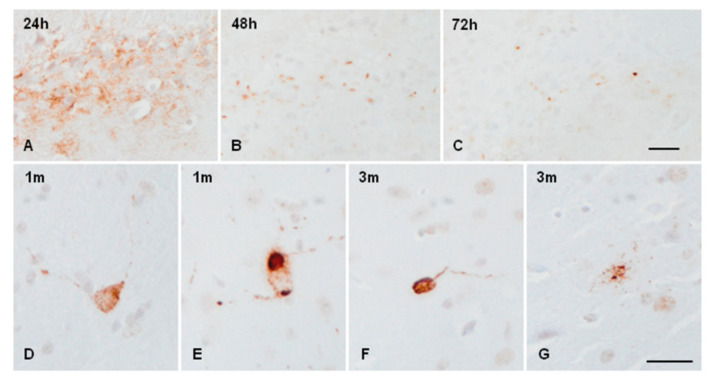
Phospho-tau immunoreactive deposits, as revealed with the antibody AT8 in mice unilaterally inoculated with sAD sarkosyl-insoluble fractions in the ventral thalamus at the age of newborn and killed 24 h, 48 h, 72 h, 1 month, and 3 months later. Diffuse AT8 immunoreactivity is seen at the injection site after 24 h (**A**). Positive dots are seen at the injection site 48 h (**B**) and 72 h after inoculation (**B**,**C**). Very rarely, isolated positive neurons with granular cytoplasm and fine radiating dendrites and exceptional dense cytoplasmic inclusions are seen in mice aged 1 month (**D**,**E**,**G**) and 3 months (**F**). Paraffin sections slightly counterstained with haematoxylin, bar = 25 µm.

Diffuse AT8-positive staining at the site of the injection was observed 24 h after inoculation (Figure 4A). Small phospho-tau-immunoreactive dots, as revealed with the AT8 antibody, were seen at the site of the injection of sarkosyl-insoluble fractions 48 h and 72 h after inoculation (Figure 4B,C). Very small numbers of neurons containing phospho-tau were observed at 1 month (Figure 4D,E); several serial sections were needed to see a single positive neuron per section, if any, at the age of 3 months (Figure 4F,G). AT8-immunoreactive neurons were distributed in the ipsilateral ventral lateral, ventral posterolateral, and ventral posteromedial thalamic nuclei. Positive neurons showed fine granular tau-immunoreactive deposits in the cytoplasm and proximal dendrites (Figure 4D,F,G); round and dense aggregates were only exceptionally observed (Figure 4E). No tau-immunoreactive neurons were seen in mice killed at the age of six months. No tau-immunoreactive neuronal inclusions were identified in mice inoculated with sarkosyl-soluble fractions at the newborn age and killed at the age of 1 month (data not shown). Common reactions in inoculated mice were the presence of phagocytes during the first week and the presence of a few reactive astrocytes along the trajectory of the needle at the age of one month (data not shown). 

### 2.4. Inoculation of Sarkosyl-Insoluble and Sarkosyl-Soluble Fractions from sAD into the Thalamus of Adult WT Mice (Group 3)

**Figure 5:**
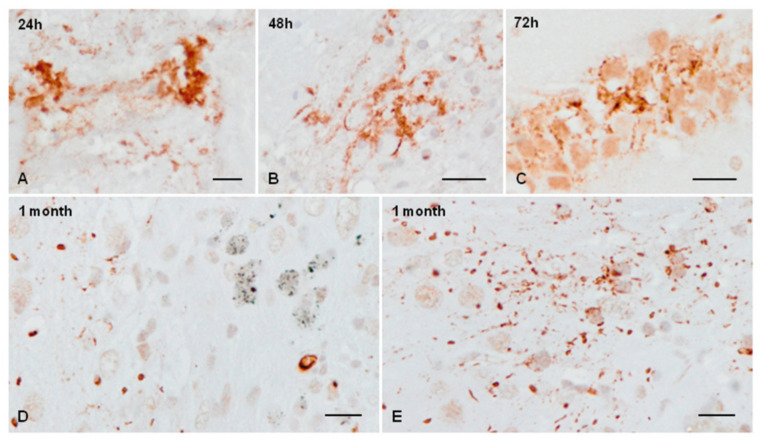
Phospho-tau immunoreactive deposits, as revealed with the antibody AT8 in mice unilaterally inoculated with sAD sarkosyl-insoluble fractions in the ventral thalamus at the age of 3 months and killed 24 h, 48 h, 72 h, 1 month, and 3 months after injection. Diffuse AT8 immunoreactivity is seen at the injection site after 24 h (**A**). Tau-immunoreactive local dot-like and fine thread deposits are localised around the cytoplasm of cells at 48 h (**B**) and 72 h (**C**). Positive dots, threads, and isolated cells show AT8 immunoreactivity at a distance from the injection site one month after inoculation (**D**,**E**). Paraffin sections slightly counterstained with haematoxylin, bar = 25 µm.

WT mice inoculated with sarkosyl-insoluble fractions at the age of 3 months and killed 24 h later showed diffuse AT8 immunoreactivity at the inoculation site (Figure 5A). Phospho-tau-immunoreactive granules were observed surrounding the membrane of local neurons at 48 h and 72 h after inoculation (Figure 5B,C). Small granules, threads, and positive cytoplasmic inclusions were identified at some distance from the injection site 1 month after inoculation (Figure 5D,E). 

**Figure 6:**
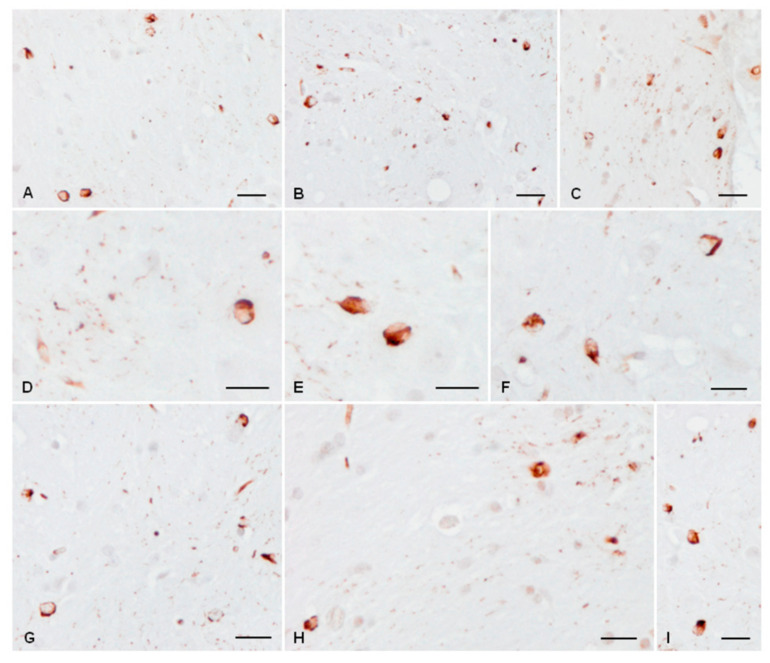
Large numbers of phospho-tau deposits, as revealed with AT8 antibody, in cells and threads in different thalamic nuclei of mice unilaterally inoculated with sAD sarkosyl-insoluble fraction in the ventral thalamus at the age 3 months and killed at the age of 6 months (**A**–**D**) or 9 months (**E**–**I**) (**A**–**I**). Tau-immunoreactive inclusions are round or elongated deposits in the cytoplasm and fine neurites in the neuropil (threads); proximal dendrites do not contain phosphorylated tau. Paraffin sections slightly counterstained with haematoxylin, bar = 25 µm.

Inoculated mice at the age of 3 months and killed at the age of 6 months (Figure 6A–D) or 9 months (Figure 6E–I) showed numerous cells with cytoplasmic AT8-immunoreactive deposit and threads in the ipsilateral ventral lateral, ventral posterolateral, ventral posteromedial, lateral dorsal, and reticular nuclei of the thalamus. In addition, AT8-positive neurons were observed in the habenula and caudate/putamen, together with positive fibres and glial cells with tau-positive inclusions in the internal capsule and fimbria (Figure 6).

No AT8-immunoreactive deposits were observed in the somatosensory cortex, hippocampal complex, amygdala, or hippocampus. Similar deposits were seen in mice inoculated with sarkosyl-insoluble fractions and killed at the age of 9 months (6 months’ survival). No tau deposits were found in mice inoculated with sAD sarkosyl-soluble fractions. Reactive astrocytes and microglia were present along the trajectory of the needle in mice inoculated with sAD fractions and with vehicle alone; a few macrophages were clustered at the inoculation site, and a few reactive astrocytes and residual macrophages were still present in some cases months after the inoculation. In no case did microglial or astroglial responses extend beyond the restricted territory of the traumatic lesion produced by the needle. 

### 2.5. Brain Inoculation of sAD Sarkosyl-Insoluble Fractions in Newborn Mice Does Not Prevent Tau Seeding following Re-Inoculation of sAD Sarkosyl-Insoluble Fractions at the Age of Three Months (Group 4)

**Figure 7:**
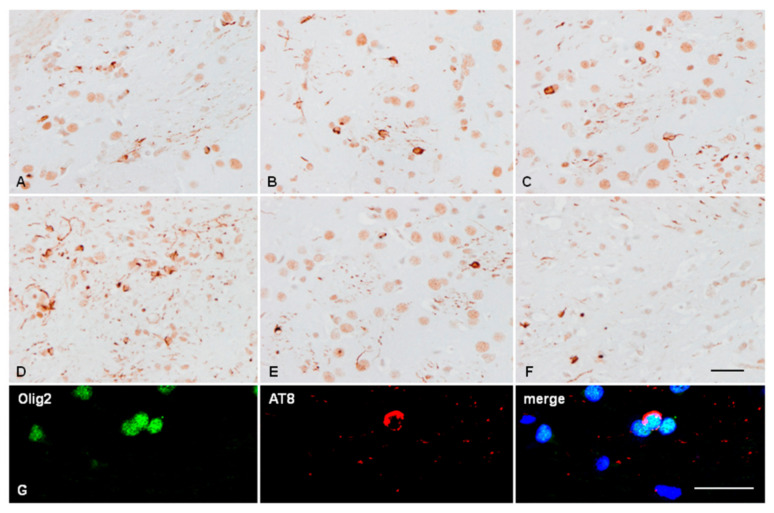
Large numbers of AT8-positive neurons, glial cells, and threads in different thalamic nuclei in mice inoculated in the ventral thalamus with sAD sarkosyl-insoluble fractions at the age of newborn and then re-inoculated with sAD sarkosyl-insoluble fractions in the thalamus at the age of 3 months. Mice were killed at the age of six months (**A**–**C**) or 9 months (**D**–**F**). Paraffin sections slightly counterstained with haematoxylin, bar = 50 µm. Double-labelling immunofluorescence with AT8 and Olig2 shows localization of tau deposits in oligodendroglia. Paraffin sections, nuclei stained with DRAQ5TM, bar = 20 µm (**G**).

Newborn mice inoculated with sarkosyl-insoluble fractions into the thalamus, re-inoculated in the ipsilateral thalamus with sAD sarkosyl-insoluble fractions at the age of 3 months, and killed at the age of 6 months showed large numbers of cells with tau-immunoreactive deposits in the cytoplasm. Positive cells were distributed in the ipsilateral ventral lateral, ventral posterolateral, ventral posteromedial, lateral dorsal, and reticular nuclei of the thalamus; positive cells were also observed in the caudate/putamen and corpus callosum (Figure 7A–F). Since some cells had the appearance of glial cells, double-labelling immunofluorescence and confocal microscopy with AT8 and Olig2 or GFAP were used for assessment. Tau-positive deposits were localised in oligodendrocytes (Figure 7G). Tau deposits were not seen in GFAP-immunoreactive astrocytes (data not shown). 

**Figure 8:**
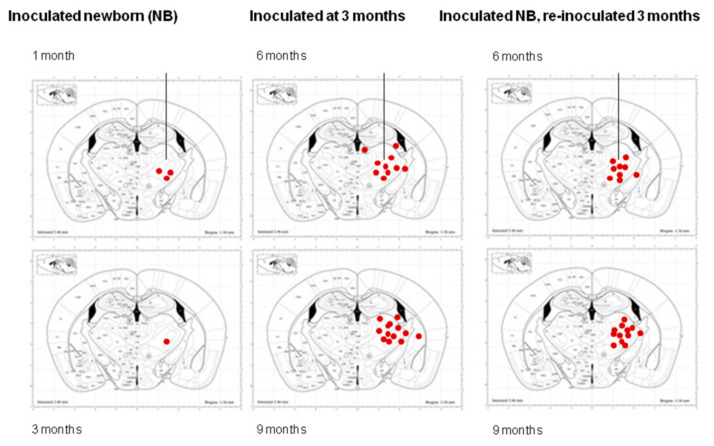
Distribution of phospho-tau deposits in mice following unilateral inoculation in the ventral thalamus with a sarkosyl-insoluble fraction from sAD in newborn mice killed at 1 month and 3 months (group 2), inoculated at the age of 3 months and killed at the age of 6 months or 9 months (group 3), and inoculated at the age of newborn, re-inoculated at the age of 3 months (group 4), and killed at the age of 6 months or 9 months. Diagrams modified from Paxinos and Franklin, 2019 (see Methods section). In inoculated newborn mice, a few AT8-containing cells are dispersed in the ipsilateral ventral lateral, ventral posterolateral, and ventral posteromedial thalamic nuclei at 1 month; tau-containing cells are barely present in mice aged 3 months. In contrast, large numbers of tau-containing cells and threads are seen in the ipsilateral ventral lateral, ventral posterolateral, ventral posteromedial, lateral dorsal, and reticular nuclei of the thalamus and in the habenula and caudate/putamen in mice inoculated at the age of 3 months and killed at the age of 6 months or 9 months. In addition, positive fibers and glial cells are seen in the internal capsule and fimbria. A similar amount and distribution of AT8-immunoreactive cells occur in inoculated mice at the age of newborn, re-inoculated at 3 months, and killed at the age of 6 months and 9 months. Similar patterns are seen in mice surviving 3 months and 6 months following inoculation. Red dots are representative of the distribution of seeding. The vertical lines indicate the injection site.

The distribution of positive cells in mice inoculated in the thalamus with sAD sarkosyl-insoluble fractions (a) at the age of newborn and killed at one month and three months; (b) at the age of three months and killed at the age of 6 months or nine months, and (c) at the age of newborn, re-inoculated at the age of three months, and killed at the age of six months or nine months, is illustrated in Figure 8. 

**Figure 9:**
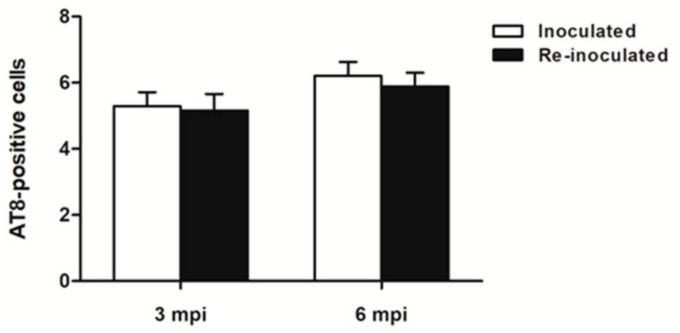
Quantification of AT8-immunoreactive cells in the thalamus in mice inoculated with sarkosyl-insoluble fraction at the age of newborn and re-inoculated at 3 months (group 4), and killed at 6 months and 9 months (3 mpi and 6 mpi: survival 3 months and 6 months, respectively), and in mice inoculated with a sarkosyl-insoluble fraction the age of 3 months (group 3) and killed 3 months and 6 months later (3 mpi and 6 mpi, respectively). No differences in the number of AT8-immunoreactive cells are seen among the four lots of mice. Values correspond to the number of positive cells in an arbitrary area of 0.045 mm^2^.

Quantitative studies of AT8-immunoreactive cells in an arbitrary area of the thalamus measuring 0.045 mm^2^ in mice inoculated at 3 months (group 3) and in mice inoculated with sarkosyl-insoluble fractions at the age of newborn and then re-inoculated at 3 months (group 4) and surviving 3 months was 5.292 ± 0.42 and 5.167 ± 0.49 (*t*-test, *p*-value: 0.843). The number of AT8-immunoreactive cells in mice surviving 6 months was 6.208 ± 0.42 and 5.889 ± 0.41, respectively, for mice inoculated at 3 months (group 3) and mice inoculated with sarkosyl-insoluble fractions at the age of newborn and re-inoculated at 3 months (group 4) (*t*-test, *p*-value: 0.5994) (Figure 9).

## 3. Discussion

Alzheimer’s disease and tauopathies are characteristically adult neurodegenerative diseases. The first neurofibrillary tangles in the human brain appear in the third decade of life in the locus coeruleus and raphe nuclei, entorhinal cortex and transentorhinal cortex, and olfactory bulb and tracts, to increase in numbers and extension to other brain regions with brain ageing and Alzheimer’s disease [4,5,6,56,57,58,59]. Hyper-phosphorylated tau occurs at the early stages of normal human brain development, but it never forms aggregates similar to those seen in tauopathies [38,39]. Tau phosphorylation has been reported in adolescents exposed to high levels of contamination compared with children from nonpolluted areas [60]. Neurofibrillary tangles in young patients are seen only in rare conditions such as Niemann–Pick’s disease type C, subacute sclerosing panencephalitis, and genetic syndromes linked to PLA2G6 and SLC9A6 mutations [2]. 

The present observations show that newborn mice are resistant to tau seeding and spreading following inoculation of sarkosyl-insoluble fractions from sAD into the thalamus. In contrast, deposits of phosphorylated tau are abundant in mice inoculated at the age of 3 months with the same inocula in the thalamus and surviving 3 and 6 months. This observation is in line with previous findings showing that old mice are more prone than young mice to tau seeding and spreading [55]. The differences are even more striking between newborn and young mice aged 3 months than those described in young versus old mice [61]. 

Protein tau in the human brain is expressed in six isoforms arising from alternative splicing of exons 2 and 3, which encode N-terminal sequences, and exon 10, which encodes a microtubule-binding repeat domain; isoforms with 352 (3R/0N), 381 (3R/1N), and 410 (3R/2N) amino acids are 3Rtau, and isoforms with 383 (4R/0N), 412 (4R/1N), and 441 (4R/2N) amino acids are 4Rtau [62]. In human and rodent fetal brains, the smallest 3Rtau isoform that lacks sequences from exons 2, 3, and 10 (3R/0N) is predominant [63,64,65,66,67]. All six isoforms are expressed in the adult human brain at a 1:1 ratio; in contrast, 4Rtau isoforms predominate in the brain of adult mice [63,64,67,68,69,70,71,72,73,74]. 

A shift from fetal to adult tau isoform expression occurs in most species, including mice and humans. This is manifested by a predominance of 3Rtau isoforms during the early stages of development and then increased 4Rtau isoforms in the adult brain, with high levels of phosphorylated tau in the developing brain when compared with the adult brain. These changes occur before the end of weaning in mice and are delayed for longer periods in humans [65,67,75,76,77]. However, subpopulations expressing 3Rtau persist in the hippocampus in the adult murine brain [78,79,80]. 3Rtau is also expressed in the olfactory bulb and periventricular regions and in a few neurons in the periventricular hypothalamus, thalamus, basal forebrain, amygdala, and cerebral cortex in adult mice. This may explain the presence of 3Rtau bands in Western blots of adult mice. 

Differential regulation of microtubule dynamics by 3Rtau and 4Rtau has been implicated in the onset of neurodegenerative diseases [81]. It can be suggested that the profile of tau isoforms during development may contribute to the reduced production of tau aggregate in the youngest. 

Unilateral inoculation of sAD sarkosyl-insoluble fractions into the thalamus in mice aged 3 months results in phosphorylated tau seeding in cells and threads in the ipsilateral ventral lateral, ventral posterolateral, ventral posteromedial, lateral dorsal, and reticular nuclei of the thalamus. Tau-immunoreactive neurons are also observed in the habenula and caudate/putamen, together with positive fibers and glial cells in the internal capsule and fimbria, thus suggesting local spreading to the striatum and habenula [82,83]. Yet differences in tau deposition are not marked when comparing survival times of 3 and 6 months after inoculation. These observations are in line with previous data suggesting reduced tau spreading in the thalamus when compared with the hippocampus [51]. Indeed, neurons of the somatosensory cortex and primary motor cortex did not contain hyper-phosphorylated tau following thalamic inoculation despite the robust connectivity between the thalamus and the somatosensory and motor cortex [84,85,86,87,88,89,90]. 

Reduced tau seeding and spreading in newborn mice compared with mice aged 3 months may also be related to the immaturity of thalamic connections at this age [91,92], in addition to seeding resistance. 

In any case, tau seeding and spreading are not accompanied by astrocytic and microglial responses. 

Brain inoculation of sarkosyl-insoluble fractions in the newborn mouse does not protect from tau seeding after intracerebral re-inoculation of the same fractions at the age of 3 months. The amount and distribution of tau deposits in re-inoculated mice are similar to those seen in mice inoculated at the age of 3 months and surviving 3 or 6 months. sAD sarkosyl-insoluble fractions contain different tau species along with many other molecules that are components of human neurofibrillary tangles [93]. Moreover, murine tau differs from human tau in a number of ways, including in the N-terminal domain (residues 18 to 28) and three amino acid residues in the C-terminal domain [62,66,68,94,95]. Despite these differences between human and murine tau, in addition to the presence of additional components in sarkosyl-insoluble fractions, there is a lack of immune responses after brain inoculation of human brain homogenates containing paired helical filaments.

## 4. Materials and Methods

### 4.1. Animals

Wild-type C57BL/6 mice from our colony were used. All animal procedures were carried out following the guidelines of the European Communities Council Directive 2010/63/EU and with the approval of the local ethical committee (C.E.E.A: Comitè Ètic d’Experimentació Animal; University of Barcelona, Spain; ref. 426/18). The animals were maintained under standard conditions of 12 h light/dark cycles, constant temperature, and free access to food and water. Newborn mice were maintained with their mothers in individual cages, one mother per cage, until weaning. The animals analysed were of both sexes, following the criteria used in our institute to avoid sex discrimination and the use of the minimal number of animals necessary for the present study. 

The following four groups of mice were assessed:

1. WT mice aged 15 days, 3 months, and 12 months of both sexes were used for Western blot (n = 4 for every time point; total 12) and immunohistochemical studies (n = 6 per age, total = 18). Twelve animals of the eighteen were used only for Western blotting;
2. Newborn WT mice aged 1–5 days were inoculated in the right thalamus with sarkosyl-insoluble fractions of brain homogenates from sAD and killed at the following times postinoculation: 24 h (n = 2), 48 h (n = 2), 72 h (n = 2), 1 month (n = 3), 3 months (n = 6), and 6 months (n = 4). In addition, four newborn mice aged 5 days were inoculated with sarkosyl-soluble fractions from sAD and killed at the age of 1 month. The total number of mice was 23, indistinctly males or females;
3. WT mice aged 3 months were inoculated into the right thalamus with sarkosyl-insoluble fractions of brain homogenates from sAD and killed at the following times after inoculation: 0 h (n = 2), 24 h (n = 2), 48 h (n = 2), 72 h (n = 2), 7 days (n = 2), 3 months (n = 4), and 6 months (n = 4) after inoculation. Four mice aged 3 months were inoculated with sarkosyl-soluble fractions from sAD, and killed at the age of 6 months (survival 3 months). The total number of mice was 22, including equal numbers of males and females. In parallel, two WT mice aged 3 months were injected with 50 mM Tris-HCl (pH 7.4) as vehicle (negative) controls;
4. Newborn WT mice aged 1–5 days (n = 6) were inoculated in the right thalamus with sarkosyl-insoluble fractions of brain homogenates from sAD, and re-inoculated in the right thalamus with sarkosyl-insoluble fractions of sAD at the age of 3 months. Mice were killed at the age of 6 months (survival 3 months) or 9 months (survival 6 months). The total number of mice in this group was six.

### 4.2. Western Blotting of Total Mouse Brain Homogenates of Noninoculated Mice

Noninoculated WT mice aged 15 days, 3 months, and 12 months (n = 4 for every time-point) (group 1) were killed under anaesthesia, and the brains were rapidly removed from the skull. The left hemisphere was immediately frozen on dry ice and stored at −80 °C until used for Western blot studies. The right hemisphere was rapidly fixed with 4% paraformaldehyde in phosphate buffer and embedded in paraffin for immunohistochemistry.

Brain homogenates were lysed in RIPA buffer (50 mM Tris-HCl, pH 7.0; 150 mM NaCl, 1% Nonidet P-40; 0.5% Na-deoxycholate; 0.1% SDS) supplemented with protease and phosphatase inhibitors (Roche, Basel, Switzerland). After centrifugation at 20,000× *g* for 20 min at 4 °C (Ultracentrifuge Beckman with 70Ti rotor, Barcelona, Spain), supernatants were quantified with BCA reagent (Pierce, Waltham, MA, USA). Protein samples were mixed with loading sample buffer and heated at 95 °C for 5 min. 20 µug of protein was separated by electrophoresis in SDS-PAGE gels and transferred to nitrocellulose membranes (200 mA per membrane, 120 min). Nonspecific binding was blocked by incubation in 5% nonfatty milk in Tris-buffered saline (TBS) containing 0.2% Tween (TBS-T) for 1 h at room temperature. After washing, the membranes were incubated at 4 °C overnight with one of the primary antibodies (Table 1) in TBS containing 3% albumin and 0.2% Tween. Membranes were washed with TBS-T and incubated for 1 h at room temperature with the appropriate horseradish peroxidase-conjugated secondary antibody (1:2000; Dako, Glostrup, DE). Immune complexes were revealed by incubating the membranes with a chemiluminescence reagent (Electrochemiluminescence; Amersham, GE Healthcare, Buckinghamshire, UK). Densitometry was carried out with Totallab software (TL100 v.2006b), and values were normalised using β-actin. The normality of distribution of fold change values was analysed with the Kolmogorov–Smirnov test. The Unpaired *t*-test was used. Statistical analysis and graphic design were performed with GraphPad Prism version 5.01 (La Jolla, CA, USA). The data were expressed as mean ± SEM, and significance levels were set at *p* < 0.05, *p* < 0.01, and *p* < 0.001.

### 4.3. Tissue Processing for Immunohistochemistry and Immunofluorescence

Group 1 WT mice aged 15 days (n = 6), 3 months (n = 6), and 12 months (n = 6), total n = 18, and group 2 (n = 23), group 3 (n = 25), and group 4 (n = 6) inoculated mice were killed under anaesthesia, and the brains were rapidly fixed with 4% paraformaldehyde in phosphate buffer, and embedded in paraffin. Consecutive serial sections 4 μm thick were obtained with a sliding microtome. Dewaxed sections were stained with haematoxylin and eosin or processed for immunohistochemistry with antibodies listed in Table 1. Following incubation with the primary antibody, the sections were incubated with EnVision + system peroxidase for 30 min at room temperature. The peroxidase reaction was visualised with diaminobenzidine and H_2_O_2_. Control of the immunostaining included omission of the primary antibody; no signal was obtained following incubation with only the secondary antibody. Quantification of AT8-positive cells in the thalamus was carried out in inoculated mice aged 3 months (group 3) and in newborn inoculated mice re-inoculated at the age of 3 months (group 4) with sarkosyl-insoluble fractions. Counts were made at survival times of 3 months and 6 months (n = 8 and n = 6, respectively, for group 3 and group 4) in random areas of 0.045 mm^2^ in every case. Values were expressed as mean values ± SEM. The *t*-test was used to assess differences between paired groups. 

Double-labelling immunofluorescence was carried out on dewaxed sections, 4 μm thick. The sections were boiled in citrate buffer to enhance antigenicity and blocked for 30 min at room temperature with 10% fetal bovine serum diluted in 0.1 M phosphate-buffered saline (PBS). The sections were stained with a saturated solution of Sudan black B (Merck, Kenilworth, NJ, USA) for 15 min to block autofluorescence of putative lipofuscin granules present in cell bodies and then rinsed in 70% ethanol and washed in distilled water. Then the sections were incubated at 4 °C overnight with AT8 and rabbit polyclonal Olig2, or rabbit polyclonal anti-GFAP or Iba1. After washing, the sections were incubated with Alexa488 or Alexa546 fluorescence secondary antibodies against the corresponding host species. Nuclei were stained with DRAQ5TM. Then the sections were mounted in Immuno-FluoreTM mounting medium (MP Biomedicals, CA, USA), sealed, and dried overnight. Sections were examined with a Leica TCS-SL confocal microscope. Identification of brain regions was made following Paxinos and Franklin, 2019, and Schröder et al., 2019 [96,97].

### 4.4. sAD Sarkosyl-Insoluble and Soluble Fractions Used for Brain Inoculation 

Samples of the frontal cortex (10 g) from one man, aged 68 years, with sporadic AD (sAD) stage VI/C of Braak and Braak and phase 4 of Thal without comorbidities were obtained 4 h postmortem and immediately frozen at −80 °C until use. The samples were provided by the Institute of Neuropathology Brain Bank, now a branch of the HUB-ICO-IDIBELL Biobank, following the guidelines of the Real Decreto 1716/2011 of the Spanish legislation and the approval of the local ethics committee. The amount of 10 g of the brain tissue was lysed in 10 vol ) with cold suspension buffer (10 mM Tris-HCl, pH 7.4, 0.8 M NaCl, 1 mM EGTA) supplemented with 10% sucrose, protease, and phosphatase inhibitors (Roche, Basel, Switzerland). The homogenates were centrifuged at 20,000× *g* for 20 min (Ultracentrifuge Beckman with 70Ti rotor,) and the supernatant (S1) was saved. The pellet was rehomogenised in a 5 vol buffer and recentrifuged at 20,000× *g* for 20 min. The two supernatants (S1 + S2) were mixed and incubated with 0.1% N-lauroylsarkosynate (sarkosyl) for 1 h at room temperature while being shaken. Samples were then centrifuged at 100,000× *g* for 1 h. Sarkosyl-insoluble pellets were resuspended (0.2 mL/g) in 50 mM Tris-HCl (pH 7.4). Protein concentrations were quantified with the bicinchoninic acid assay (BCA) assay (Pierce, Waltham, MA, USA). Sarkosyl-insoluble fractions were processed for Western blotting. Samples were mixed with loading sample buffer and heated at 95 °C for 5 min. 60 µug of protein was separated by electrophoresis in SDS-PAGE gels and transferred to nitrocellulose membranes (200 mA per membrane, 90 min). The membranes were blocked for 1 h at room temperature with 5% nonfat milk in TBS containing 0.2% Tween and were then incubated with the phospho-specific antibody anti-tau Ser422 (diluted 1:1000; Thermo Fisher, Waltham, MA, USA). After washing with TBS-T, blots were incubated with anti-rabbit IgG conjugated with horseradish peroxidase (diluted at 1:2000; Agilent, Santa Clara, CA, USA) for 45 min at room temperature. Immune complexes were revealed by incubating the membranes with a chemiluminescence reagent (Amersham, Germany Healthcare, Life Sciences, Buckinghamshire, UK) [55]. Western blotting of sarkosyl-soluble fractions included, in addition to anti-tau Ser422, the incubation with anti-4Rtau and anti-3Rtau antibodies (dilutions 1:1000 and 1:2000, respectively) to control the presence of tau.

In addition, sarkosyl-insoluble and sarkosyl-soluble fractions were analyzed with transmission electron microscopy (TEM) procedures and thioflavin T (ThT) amyloid quantification assay. Sarkosyl-insoluble fractions were fixed to carbon-forward-coated copper supports, and negative staining was performed using 2% uranyl acetate (pH 7.4). The samples were then placed in silica-based desiccant for a minimum of 2 h and examined with a Jeol JEM-1010 transmission electron microscope. ThT stock solution was prepared at 2.5 mM (dissolved in 10 mM phosphate buffer, 150 mM NaCl, pH 7.0) and preserved in a single-aliquot at −80 °C. The ThT assay was performed by dissolving 0.2 µL of sarkosyl-insoluble fraction in 0.2 mL of freshly prepared ThT (final concentration 30 µM), followed by quantification using an absorbance/excitation (445/485) microplate reader (Tecan Infinite M200Pro, Männedorf, Switzerland) in 96 flat-bottom polystyrol plates (Nunclor, ThermoFisher Scientific, Germany). Plates were prepared and incubated at 37 °C. Readings were taken each hour over the 15–16 h period.

### 4.5. Brain Inoculation of sAD Sarkosyl-Insoluble, Sarkosyl-Soluble Fractions, and Vehicle Alone 

Newborn mice aged 1–5 days were anesthetised on a cold plate surface and fixed manually. Intrathalamic injections were administered using a Hamilton syringe. A volume of 1.2 µL (amount of protein, about 3.5 µg/µL) was injected at a rate of 0.05 µL/min. The syringe was withdrawn slowly over a period of 10 min to avoid leakage of the inoculum. Following surgery, the animals were kept in a warm blanket and monitored until they recovered and then returned to the cages with their mother. Mice aged 3 months were deeply anaesthetised by intraperitoneal ketamine/xylazine/buprenorphine cocktail injection and placed in a stereotaxic frame after assuring lack of reflexes. Intracerebral injections were administered using a Hamilton syringe. The coordinates for thalamic injections were −1.3 mm AP, −1.2/−1.4 mm ML relative to Bregma (interaural 2.46 mm), and −3/−3.5 mm DV from the dural surface [96]. A volume of 1.5 µL was injected at a rate of 0.05µL/min. The syringe was withdrawn slowly over a period of 10 min to avoid leakage of the inoculum. Following surgery, the animals were kept in a warm blanket and monitored until they recovered from the anaesthesia. Carprofen analgesia was administered immediately after surgery and once a day during the two following days. Animals were housed individually with full access to food and water. 

## 5. Conclusions

Our study concludes that: (i) tau seeding following intracerebral inoculation of sAD PHFs in newborn mice is very limited in contrast to the widespread tau seeding following intracerebral inoculation of the same inocula in mice aged 3 months; (ii) differences in tau seeding between newborn and young adults may be related to the ratios between 3Rtau and 4Rtau and the shift to 4Rtau predominance in adults, together with the immaturity of connections in newborn mice; and (iii) intracerebral inoculation of sAD PHFs in newborn mice does not protect from tau seeding following intracerebral inoculation of sAD PHFs in young/adult mice. 

## Figures and Tables

**Table 1:** Antibodies used for Western blotting (wb) and immunohistochemistry (ih) in the present study. Dil—dilution.

Antibody	Reference	Supplier	Host	Dil wb	Dil ih
β-actin	A5316	Sigma-Aldrich (St Louis, MO, USA)	Ms	1:30,000	-
Tau 5	MA5-12808	Thermo Fisher (Waltham, MA, USA)	Ms	1:1000	1:100
3RTau	05-803	Millipore (Darmstadt, Germany)	Ms	1:2000	1:800
4RTau	05-804	Millipore (Darmstadt, Germany)	Ms	1:1000	1:50
P-tau Thr181	11107	Signalway (College Park, MA, USA)	Rb	1:1000	1:50
P-tau AT8	MN1020	Invitrogen, Thermo Fisher (Waltham, MA, USA)	Ms	1:250	1:50
P-tau Thr231	577813	Calbiochem (Darmstadt, Germany)	Rb	1:1000	1:50
GFAP	GA-524	Dako (Glostrup, Denmark)	Rb	-	1:500
Olig2	Ab109186	Abcam (Cambridge, UK)	Rb	-	1:500
Iba1	019-19741	Wako (Richmond, VA, USA)	Rb	-	1:1:1000

## Data Availability

All the data are in the text.
